# How to Achieve Carbon Neutrality: From the Perspective of Innovative City Pilot Policy in China

**DOI:** 10.3390/ijerph192416539

**Published:** 2022-12-09

**Authors:** Lina Liu, Yunyun Zhang, Bei Liu, Pishi Xiu, Lipeng Sun

**Affiliations:** 1 Business School, Shandong Normal University, Jinan 250358, China; 2 China Institute for Tax Governance, Shandong Normal University, Jinan 250358, China; 3 School of Management, Nanjing University of Posts and Telecommunications, Nanjing 210003, China; 4 School of Management, Wenzhou Business College, Wenzhou 325035, China

**Keywords:** synergistic development, innovative city pilot policy, carbon emissions efficiency, difference-in-differences, spatial spillover

## Abstract

The innovative city pilot policy is a new engine to accelerate the social development of China, which is an important support feature for realizing sustainable economic development. Using the city pilot policy issued by the Chinese government in 2008 as a quasi-natural experiment and the method of multi-period difference-in-differences (DID) model, we explore the effect of the policy on regional carbon emission efficiency. The research shows that the innovative city pilot policy could lead a significant promotion of the carbon emission efficiency of cities, which shows the characteristics of dynamic sustainability, that is, the policy effect continues to increase over time. Mechanism analysis reveals that the innovative city pilot policy mainly drives the improvement of urban carbon emission efficiency through improving the green technology innovation level of pilot cities, promoting the upgrading of regional industrial structure and increasing government investment in science and technology. In addition, the innovative city pilot policy has a spatial spillover effect on urban carbon emission efficiency, that is, the innovative city pilot policy not only promotes the local carbon emission efficiency, but also improves the carbon efficiency of neighboring areas.

## 1. Introduction

Due to the dramatic change in China’s economy over the past few decades, environmental problems such as climate warming have accelerated [1]. Climate warming, caused by excessive carbon dioxide emissions, has severely threatened global economic development [2,3,4]. In 2017, Xi Jinping clearly pointed out for the first time in his report to the 19th National Congress that the primary task in the new stage of development is to build a new paradigm that wins with quality. Nowadays, China’s carbon emissions are still in the historic period of “high total amount and high increment”. By the end of 2020, the ratio of fossil energy used in China is about 85%, which is relatively high. According to the 2020 Global Environmental Performance Index Report released by Yale University, the environmental problems are still severe in China. To transform the high energy consuming economic development model, China actively promotes the construction and development of innovative cities. By implementing the pilot policy which helps cities establish and improve innovative development mechanisms, China is attempting to promote urban transformation and upgrading, build new innovative cities, guide and finally form a development model that is green and environmentally friendly to drive the development of innovative cities across the country.

China’s continued economic growth has provoked serious environmental problems [5]. Cities, which are not only spatial carriers of human activities, but also centers of socio-economic development [6], have long been recognized as major engines of social innovation and wealth creation, having an inextricably linked relationship with urban development and economic innovation [7]. The existing literature indicates that factors affecting the process of carbon neutrality mainly include energy consumption [8,9], intelligent industry [10,11], economic development [12,13] and energy structure [14,15], environmental regulation [16,17], building construction [18], etc. Urbanization is also closely related to carbon emissions. On the one hand, urbanization will increase resource consumption and lead to excessive carbon dioxide emissions [19,20]; on the other hand, carbon emissions can also be reduced through industrial upgrading and technological progress [21]. With 70% of the world’s carbon emissions produced by cities, low-carbon urban development is crucial. Although cities are important carriers of economic development and human civilization, they consume more than 60 percent of fossil energy and generate more than 70 percent of human carbon emissions. The realization of carbon peaking and carbon neutrality is inseparable from the development of technological innovation. Innovation is the primary driving force for development, and science and technology are powerful tools to solve environmental problems. Then, as an important measure to improve the level of urban innovation, does the innovative city pilot policy improve the efficiency of urban carbon emission? Based on this logic, we take the pilot policy as the origin and concentrate on the pollution and emission reduction effect of the government actively carrying out pilot policies. Has the policy prompted the performance of urban carbon emissions since its implementation? What is the mechanism underlying the improvement? Is there a space effect on the carbon emission efficiency of adjacent areas? It is of great practical relevance for the expansion of innovative cities and future development to identify the above problems accurately and objectively, which provides policy enlightenment for China to achieve better development.

The marginal contributions of this paper are as follows: (1) Based on the data of 281 prefecture-level cities in China from 2006 to 2019, this paper examines the impact of innovative city pilot policies on carbon emission efficiency by using the multi-period differential method. (2) The different contributions of green technology innovation, industrial structure upgrading and government S&T investment path to the improvement of carbon emission performance are comprehensively investigated. (3) Considering the possible spatial spillover effect of carbon emission efficiency and its determinants, this paper uses the spatial difference-in-differences (SDID) model to test the impact of pilot policies of innovative cities on neighboring carbon emission efficiency. The remaining structure of this paper is arranged as follows: the second part is the policy background and literature review, the third part describes the research design, the fourth part conducts the empirical structure and analysis, the fifth part presents the spatial effect analysis, and the sixth part discusses the conclusion and revelation.

## 2. Policy Background and Literature Review

### 2.1. Policy Background

With the continuously strengthened financial support in science and technology, the scientific and technological capabilities of China have been continuously enhanced. However, compared with the developed countries in the Western economy, there is still a big gap, a lack of independent innovation capabilities and insufficient development of core innovative technologies. Transforming the traditional mode of economic development to promote innovation-driven development is a tremendous driver of urban economic development [22] and an important measure to achieve sustainable development [23]. To achieve green development, the Chinese authority is striving to enhance the innovation capabilities of cities. The strategic plan for an innovative country was first formally proposed by the State Council in 2006, under which local governments at all levels responded and suggested building innovative cities. The application of the innovative pilot policy is of great importance to China’s urban construction. It is an objective requirement of cultivating new momentum for economic development and achieving economic leapfrog development, and an important measure to increase China’s rank and push the country to the forefront of innovative countries.

**Figure 1:**
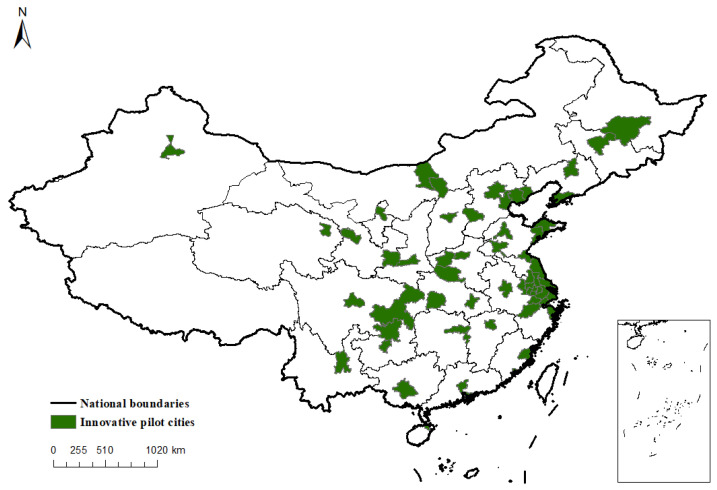
Distribution of Innovative Pilot Cities in China.

To prepare for a smooth construction of innovative pilot cities, the NDRC approved Shenzhen as a national innovative pilot city for the first time in June 2008. In January 2010, the NDRC issued the Notice on Promoting the Pilot Work of National Innovative Cities, which approved 36 cities, including Dalian, as national innovative pilot cities, and the process of constructing innovative cities entered a stage of large-scale trials. Since then, the scope of innovative pilot cities has been progressively expanded, and as of 2013, there have been 58 innovation pilot cities. By continuously expanding the scope of innovative pilot cities, China actively encourages innovative pilot cities to explore urban innovation and development paths so as to build regional innovation demonstration cities. It is foreseeable that the execution of the innovative pilot policy will make a significant difference to the economic development of China. Figure 1 shows the spatial distribution of innovative pilot cities in China.

### 2.2. Theoretical Analysis and Hypothesis Formulation

This paper is related to three principal fields: first, the relativity between the innovative city pilot policy and carbon efficiency; second, the internal mechanism to improve the urban carbon emission efficiency, and finally, the spatial spillover effect of the pilot policy.

#### 2.2.1. The Pilot Policy and Carbon Efficiency

Under the tremendous pressure of China’s economic downturn and deteriorating ecological conditions, innovation is undoubtedly the key to achieving harmonious development [24]. There is little research on trials of innovative cities, which mainly focuses on the urban innovative and developing level of cities, environmental performance and knowledge innovation. Taking cities in China as study subjects, an evaluation of the current situation of innovative city construction is assessed by Fang et al. [25], indicating that the level of the innovation in Chinese cities is still low, and there remains a huge disparity between China’s cities and those of developed countries. Under the premise of expanding city scale and deepening innovation concept, innovative city pilot is a major initiative of China’s urban development and innovation [26]. Cao et al. [27] argue that the urban innovation strategy has a favorable impact on innovation. Fan et al. [28] verify that increased efficiency of urban innovation significantly improves haze pollution in cities, and that increased levels of urban innovation can effectively reduce environmental risks [29] and achieve sustainable urban economic development [30]. By studying the effect of innovative city pilots on knowledge innovation and knowledge conversion using spatial difference models, Zhang and Wang [31] argue that innovative pilot cities can simultaneously prompt both knowledge innovation and knowledge conversion efficiency [32].

Urban development is tightly linked to carbon dioxide emissions [33], and academic research on carbon dioxide emissions is relatively rich, focusing mostly on the main factors affecting the urban carbon dioxide emissions. Taking carbon efficiency data from 30 Chinese provinces, Pan and Ming [1] indicate that a city has poor carbon performance when its proportion of heavy industry is high and its proportion of coal consumption is large. In addition, the study of Wang et al. [34] also presents an inverse correlation between resource richness and carbon efficiency, implying that resource dependence is an indirectly harmful feature of carbon efficiency by obstructing the rationalization and progress of industrial structure. However, China’s overall carbon efficiency remaining relatively poor [35] and increasing technology levels are important factors to improve carbon efficiency. As for influencing factors, carbon emission efficiency is significantly positively correlated with its level of industrial structure while it is negatively correlated with energy intensity. After exploring the variation effect of China’s carbon market on carbon dioxide emission reduction, Fan et al. [36] suggest that it has effectively improved the efficiency of carbon dioxide emission reduction, which is similar to what is argued by Zheng et al. [37], whose empirical results show that per capita carbon emissions are no longer significant due to improved energy efficiency and reduced costs associated with carbon reduction. Ang [38] argues that Rand D investment, technology application and the ability to learn from foreign technologies have adverse effect on carbon emissions in China, and higher energy use, higher incomes and higher levels of trade openness will exacerbate carbon dioxide emissions. Chu et al. [39] argue that China’s smart city policy is an inherent strength of urban innovation, and that the city’s overall innovative technological advancement enhances carbon efficiency [40].

The pilot policy is one of the actions of China to direct the growth of innovative cities, and the increase in the level of innovation is a vital initiative for the improvement of the urban environment. Through reviewing the literature, the authors of this paper believe that under the guidance of innovating cities, the government will further improve the construction of urban green infrastructure by taking actions such as building an innovation platform, providing tax relief and government subsidies to stimulate the enthusiasm of enterprises to innovate, promote cleaner production, improve energy efficiency, and reduce pollution emissions. Therefore, it seems reasonable to propose the hypothesis as follows:

**H1:**  *Innovative urban construction has a favorable impact on improving urban carbon efficiency*.

#### 2.2.2. Mechanism Analysis of the Pilot Policy to Improve Carbon Efficiency

**Figure 2:**
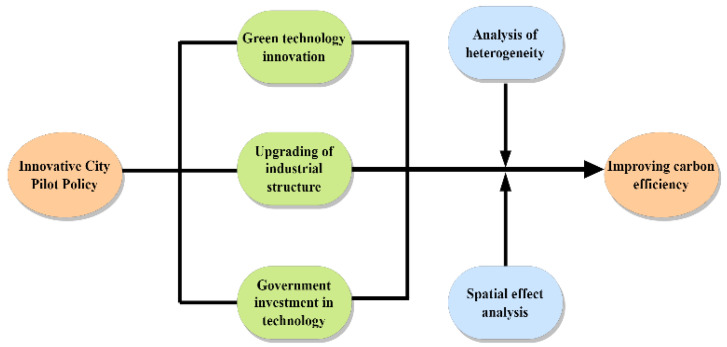
Mechanism analysis of the pilot policy to improve carbon efficiency.

Since the first implementation in 2008, the innovative city pilot work has been carried out in an orderly manner across the country. In the notice of Promoting the Pilot Work of National Innovative Cities, the NNDRC clearly proposes to enhance the city’s innovative development capacity, realize better and faster transformation of economic development model, increase the financial support in science and technology education and build a talent team, and coordinate the optimizing of urban industries. According to the above policy requirements and the review of the existing literature, this paper proposes the inherent mechanism of innovative city construction to improve carbon efficiency, and Figure 2 presents the theoretical analysis framework of the ways in which innovative city pilot policies affect carbon efficiency.

First, innovative city construction improves the urban carbon emission efficiency through technological effects. Porter and Linde [41] analyzed the effect of technology progress on environmental act in enterprises from micro perspective. Government environmental regulation policies are able to force the innovate mechanisms of companies to enhance the pollution control equipment, improve energy efficiency, and improve overall carbon emission efficiency by improving production technology. Wang and Li [42] quantitatively analyze the effect of a range of socio-economic factors on carbon productivity in China. The results show that during the gradual increase in carbon productivity between 1997 and 2016 in China, the technical level proved to promote carbon productivity, while the industrial proportions have negative effects [43]. Du et al. [44] further argue that CO_2_ emissions have been significantly reduced due to the environmental technology innovation. Technological advancement is an essential factor to improve the performance of carbon emissions in China [45].

Second, innovative city construction improves the efficiency of urban carbon emissions through structural effects, that is, it improves urban carbon emissions by optimizing industrial structure. Optimizing the industrial structure is a vital approach to realize economic transformation and high-quality development [46]. The administration has clearly proposed in the document to optimize urban industries and to coordinate and promote innovation pilot work. Qiu et al. [47] argue that low-carbon cities can improve environmental performance by optimizing regional industrial structure [48]. Ma and Cao [49] demonstrate that adjusting the weight of different industries can significantly alleviate the problem of smog pollution. Cheng et al. [50] argue that due to the phenomenon of carbon emission rebound, mere technological progress by itself cannot directly reduce carbon emission intensity, but can indirectly reduce carbon emission intensity by industry structuring through techniques change. Zhou et al. [51] argue that industrial restructuring is an important part of developing low-carbon economy. In the context of industrial structure transformation, the carbon dioxide emission of a region can be effectively decreased [52].

Third, innovative city construction prompts the efficiency of urban carbon emission by increasing government financial support in technology. In the Notice on Guidelines for The Construction of Innovative Work published in 2016, the NDRC clearly emphasized that it is necessary to boost fiscal support for local government to secure guarantees for their financial support in science and technology, and increase technology subsidies to enterprises. Until now, many studies from a micro perspective have focused on the effect of government scientific investment on enterprises, arguing that government technology subsidies have improved the innovation ability of enterprises, helped guide enterprises to turn to cleaner production, and improved energy efficiency [53,54,55]. Lin and Luan [56] indicate that there is not a simple linear correlation between government financial support and the innovation efficiency of the environmental industry, and controlling government capital expenditure before the inflection point will improve industry innovation efficiency. Li et al. [30] suggest that higher green technology subsidies provided by the Chinese government will guide cleaner green technologies implemented by enterprises, which will further reduce the total amount of pollutant emissions. Specifically, governments subsidize business investments in green technology development by taking part of the cost [57]. The research of Ma et al. [58] shows that preferential tax and government subsidies are important taxation tools, which can help low-carbon city policies to better play the pilot role and promote urban low-carbon green development.

**H2:**  *The Pilot Policy improves the carbon efficiency by raising the level of green innovation in the pilot cities, upgrading the regional industrial structure, and increasing government investment in science and technology*.

#### 2.2.3. Space Emission Reduction Effects of the Pilot Policy

With the economy developing and carbon emissions deteriorating, environmental pollution in some areas may disrupt the global ecological environment through economic globalization and other spatial spillover effects. In this regard, current studies have shown that there is a space correlation between carbon efficiency rates in adjacent regions, and carbon emissions in one region are influenced by adjacent regions [59,60,61]. Wang and Li [42] argues that China’s environmental policies have a spillover effect on the green economic growth of the industrial enterprises [62]. Marbuah and Amuakwa-Mensah [63] argue that there is spatial dependence on pollutant emissions, and the pollution reduction in a city benefits from the implementation of policies in neighboring cities. Based on Regional input–output data from 2007 to 2010 in China, Meng et al. [64] argues that domestic supply chains may affect CO_2_ emission growth in other regions. Zhang and Zhang [65] illustrate the link of carbon dioxide emissions between China, the EU and the US, from which it is seen that the carbon spillover effect of EU and the US on China is higher than that of China on them. Lan et al. [66] concluded that manufacturing agglomeration has less impact on local emissions, but has adverse effects on adjacent areas. The literature above implies the following hypothesis:

**H3:**  *The innovative city pilot policy has a space effect on the carbon efficiency of neighboring areas*.

Taking data from 2006 to 2019, we explore the policy effect on the carbon emission efficiency by DID method using the innovative city pilot policy that began to be implemented in batches from 2008 as an experiment. By a series of empirical results, we demonstrate that the pilot policy has indeed prompted the carbon emission performance of cities, which remains robust after a placebo test, a propensity match score, an increase in covariate variables, and the exclusion of other policies.

## 3. Research Design

### 3.1. Data

We select the annual data of China’s cities from 2006 to 2019 as the sample to analyze after excluding that of prefecture-level cities with new, adjusted and missing data, which finally covers 281 cities. According to the list of innovative pilot cities, 58 prefecture-level cities were sorted out as treatment groups for innovative city pilots. The pilot policy is regarded as a quasi-natural experiment, and the multi-period DID model is adopted to explore the effect of innovative city construction on the carbon efficiency. The data required for this paper mainly originates from the relevant policy documents issued by the NDRC and the MST as well as the China Urban Yearbook and the China Statistical Yearbook.

### 3.2. Model

The following econometric model is set for this research:

(1) $${CO}_{2_{it}}=\alpha_{0}+\alpha_{1}DID+\alpha_{2}Control+U_{i}+\lambda_{t}+\varepsilon_{it},$$

where *i* and *t*, respectively, represent the city and year; $CO_{2_{it}}$ is defined as the carbon efficiency of the city; *Control* represents the control variables to avoid the other factors that affect the efficiency of a city’s carbon emissions; $U_{i}$ and $\lambda_{t}$ are city and year fixed effect; $\varepsilon_{it}$ is the error term; α_1_ is the standard estimator.

### 3.3. Variable

**Figure 3:**
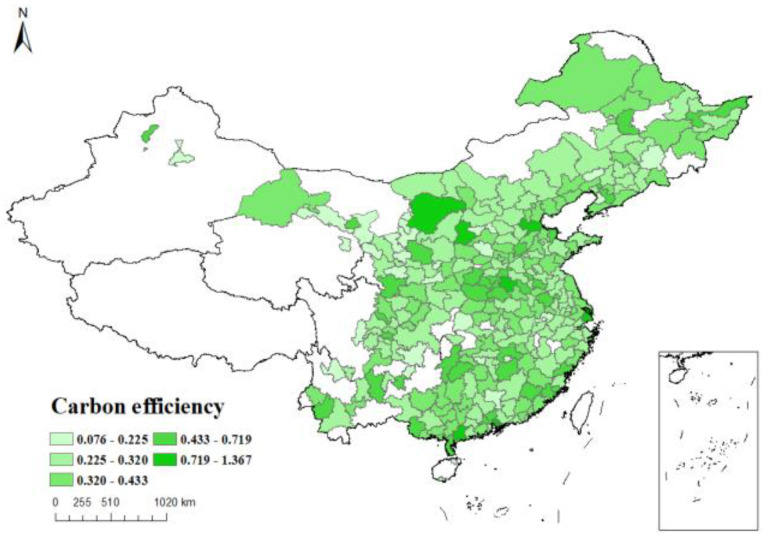
Carbon efficiency distribution of cities in China (2006).

**Figure 4:**
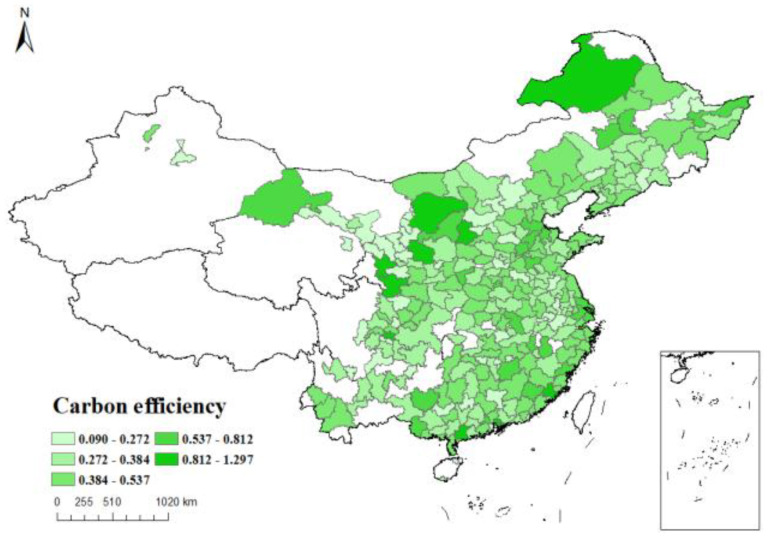
Carbon efficiency distribution of cities in China (2010).

**Figure 5:**
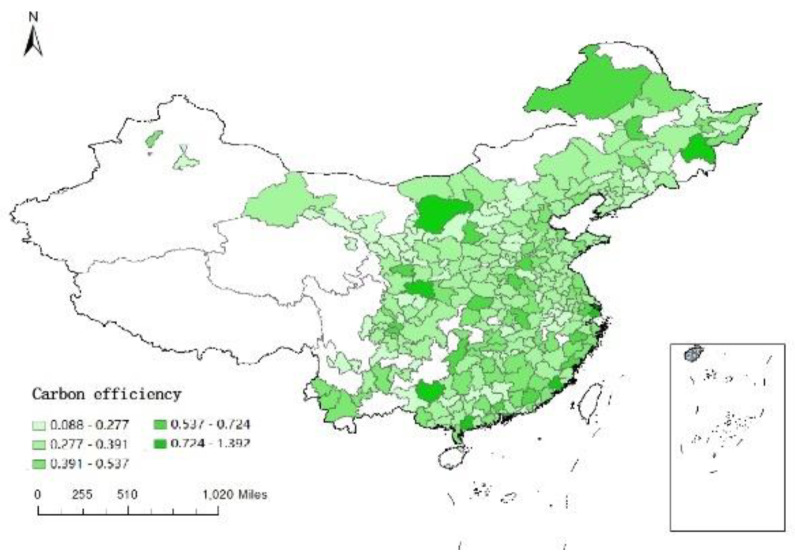
Carbon efficiency distribution of cities in China (2014).

**Figure 6:**
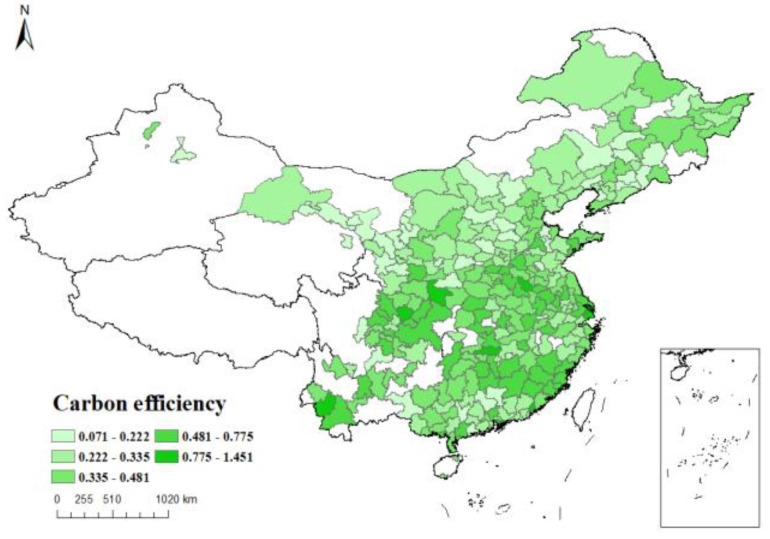
Carbon efficiency distribution of cities in China (2019).

*Dependent variable*: The natural logarithm of the carbon emission efficiency index, calculated by SBM model using data envelopment analysis, is taken as the dependent variable *Sco2*. Figure 3, Figure 4, Figure 5 and Figure 6 respectively show the carbon emission efficiency in 2006, 2010, 2014 and 2019, respectively, it can be found that the carbon efficiency of Chinese cities is generally low, and there is a wide disparity in carbon efficiency across cities. In addition, the carbon efficiency in cities has improved by a small margin over time.

*Core independent variable*: The dummy variable *DID* that takes 1 if the time is the current year or subsequent years of the implementation of the innovative pilot policy and takes 0 otherwise.

*Control variables*: To control the effects of other variables on carbon emission efficiency, referring to Yu and Zhang [67] and the others, population density (*popden*), industrial structure (*industry*), FDI proportion (*fdi*), GDP per capita (*pgdp*), and local budget revenue (*finan*) are introduced as control variables. Among them are the following: reflecting the level of urban population agglomeration, population density (*popden*), represented by logarithm of population density, is an essential factor affecting regional pollution emissions; industrial structure (*industry*) is characterized by the ratio of secondary industry value added in GDP; foreign investment level (*fdi*) is expressed in terms of the ratio of foreign investment (RMB) to regional GDP, because in the context of globalization, regional environmental problems may be transferred through patterns of economic trade; considering that the regional GDP, reflecting the overall economic development level of a region, is also a vital factor affecting urban pollution emissions, per capita gross product (*pgdp*) denoted by the natural logarithm of real per capita GDP is also introduced; local budget revenue (*finan*) is expressed in logarithmic terms of local general budget revenue. The descriptive statistical results are presented in Table 1. For Table 1, the average carbon emission efficiency is −1.104, which indicates that it is still a low level of China’s carbon emission efficiency.

## 4. Empirical Results

### 4.1. Parallel Trend Test

**Figure 7:**
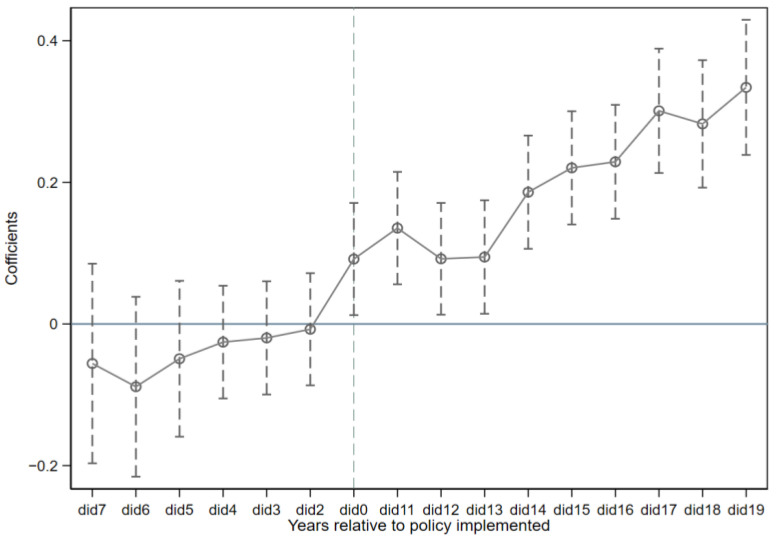
Parallel Trend Test.

The estimated results are presented in Figure 7. For *Sco2*, the regression coefficient is not significant before the policy implementation, while after the implementation it turns to a positive number that is significant, which indicates that the pilot policy improves carbon efficiency and meets the parallel trend hypothesis.

### 4.2. Baseline Regression

The baseline regression results are shown in Table 2, where column (1) shows the result without introducing any control variables or fixed effects, (2) presents the result after controlling the city fixed effect, (3) is the result after controlling the city and year fixed effects, and (4) shows the regression result taking both the control variables and fixed effect into consideration. The estimated coefficient of carbon emission efficiency of innovative cities is significantly positive in these four columns. As for the economic sense of the regression coefficient, given all other conditions are equal, the carbon efficiency of innovative pilot cities increases by 1.8% compared with non-pilot cities, which preliminarily demonstrated that the pilot policy can increase the efficiency of urban carbon emissions.

### 4.3. Robust Test

#### 4.3.1. Placebo Test

**Figure 8:**
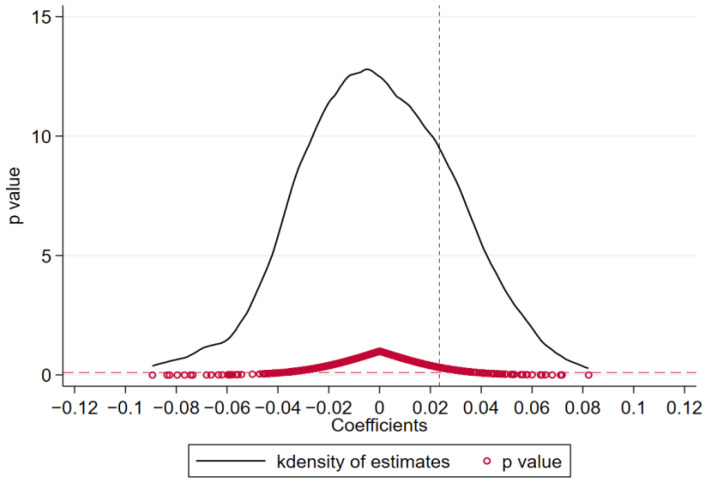
Placebo Test.

To rule out the possibility that the impact of pilot policy on carbon efficiency may be interfered with by omitted variables and exclude the impact of policy shocks that are not random, we conduct a 500-repetition placebo test shown in Figure 8 exhibiting the regression coefficient distribution plot of Sco2. As we can see from Figure 8, the regression coefficients based on random samples are distributed around 0. It can be concluded that it is a small probability event to obtain the basic regression result by accident, so the policy effect is not caused by conventional random factors or omitted variables. The figure shows that the baseline results are high probability events, and it can be considered that the policy effect is not interfered with by other factors.

#### 4.3.2. Replacing Dependent Variable

To ensure the robustness of the conclusions, we change the measurement of dependent variable and take the natural logarithm of the carbon emission efficiency index that is calculated by EBM model as the dependent variable *Eco*2, Specific empirical results are shown in Table 3 Column (4) shows the regression results taking both the control variables and the city and year fixed effects into consideration. In these four columns, the estimated coefficients of carbon emission efficiency of innovative city construction are all positive at the level of 1%, which verifies the core conclusion that the pilot policy can prompt the efficiency of urban carbon emissions.

#### 4.3.3. PSM-DID

In the process of implementing the innovative city pilot policy, it is possible that the country may choose cities with high economic development levels and strong innovation ability as the pilot city, which leads to selection bias. To overcome this bias, this paper adopts the PSM-DID method for testing, and the results are presented in Table 4. Regardless of which matching methods are used, the coefficients of *DID* are significantly positive, which confirms the core conclusion.

#### 4.3.4. Excluding Other Policies

There are inevitably some other relevant policies that may affect the estimation as shown in Table 5. Therefore, we consider three types of policy impacts that affect carbon emission efficiency, and they are the 2010 low-carbon city policy, the 2011 carbon emission trading right policy and the 2010 new energy vehicle pilot policy. By excluding pilot cities of these three types of policies, we exclude the interference of these policies and test the robustness of the conclusions. The regression coefficients of *DID* are still significantly positive, proving that the conclusion is still robust after considering the interference of other policies.

#### 4.3.5. Adding Covariates

In this paper, the intersection of control variables with both time trend third-order polynomials and time dummy variables are constructed, respectively, to control the temporal trend of factors influencing urban pollution emissions. The following formula shows our specific design ideas:

(2) $$CO_{2_{it}}=\alpha_{0}+\alpha_{1}du+\alpha_{2}dt+\alpha_{3}DID+D_{i}\left(X_{it}\times f\left(T\right)\right)+\alpha_{4}controls+\varepsilon_{it},$$

(3) $$CO_{2_{it}}=\alpha_{0}+\alpha_{1}du+\alpha_{2}dt+\alpha_{3}DID+U_{i}\left(X_{it}\times \sigma_{t}\right)+\alpha_{4}controls+\varepsilon_{it},$$

where the dependent variables $CO_{2_{it}}$ in model (2) and (3) represent the efficiency of urban carbon emissions. The $f\left(T\right)\;$ in model (2) is a third-order polynomial of the time trend, and the $\sigma_{t}$ in model (3) is a time dummy variable. The other index designs are consistent with the econometric model (1). The specific results are presented in columns (1)–(4) in the Table 6. We can determine that the coefficients of *DID* are both significantly positive at the 1% level.

#### 4.3.6. Other Robustness Tests

On the basis of the above tests, we also carried out other robustness tests, including the following three aspects: ① winsorize the statistic at 1% level; ② short the sample interval to the duration from 2008 to 2015; ③ exclude municipalities sample. The estimated results are presented in Table 7. The coefficients of *DID* are still positive at the 1% level, which again verifies the conclusion we have reached.

### 4.4. Heterogeneity Analysis

Considering that the analysis of the current sample may neglect inter-city differences, we further explore the heterogeneous effect of the pilot policy on carbon efficiency.

#### 4.4.1. Resource Endowments

According to the resource curse theory, rich natural resources lead to a high degree of regional resource dependence, while high energy dependence impedes the low-carbon transformation of economic development, which may hinder the improvement of carbon emission efficiency [68]. We divide cities into non-resource-based and resource-based cities according to the National Resource-based City Sustainable Development Plan (2013–2020), which is officially issued by the State Council, and perform regression again. The economic growth of resource-based cities mainly depends on the exploitation and utilization of fossil energy, and the industrial structure is dominated by heavy polluting enterprises. The development of non-resource-based cities depends more on the sound industrial structure and good innovation foundation to achieve high-quality development.

As we can see from Table 8, the improvement of carbon efficiency of non-resource-based city by the innovative city pilot policy is more obvious. The possible reason is that compared to resource-rich cities, the development of non-resource-based cities relies more on a sound industrial structure and a good innovation foundation, resulting in the feature that the policy performs better in non-resource-based cities and brings more green technology innovation and green development. In resource-based cities, however, the local economic growth has long been relying on natural resources. With traditional high-pollution, high-energy-consuming development model deeply rooting, it is difficult for a single innovative city pilot policy to fundamentally reverse their economic development model. Therefore, the improvement of carbon efficiency brought by the innovative pilot policy is more obvious in non-resource-based city than in resource-based city.

#### 4.4.2. Eco-Friendly Type

The 113 cities focusing on environmental protection determined by the Ministry of Environmental Protection are divided into non-environmentally friendly and environmentally friendly cities. Key cities of environmental protection reflect the pressure of the government’s environmental regulation, and compared with non-key cities of environmental protection, they may receive larger government subsidies for environmental protection and are subject to more environmental constraints in pollution control and other aspects. The two types of cities have differences in carbon emission efficiency [69]. The relevant results are presented in Table 9. From columns (1) and (2), we can observe that the regression coefficient for non-environmentally friendly cities is significantly positive at the level of 5%, while for environmentally friendly cities it is significantly positive at the level of 1%. That is, the pilot policy has a more obvious effect on improving carbon emission efficiency of environmentally friendly cities.

### 4.5. Transmission Mechanism Test

The conclusion that the pilot policy has apparently improved carbon efficiency performance has been demonstrated by the benchmark regression. The question now becomes: by what mechanism does this policy prompt the carbon emission efficiency? We analyze the three realization paths of green technology innovation, including urban industrial structure, local government science and technology expenditure.

Table 10 presents the main results. Column (2) reveals that the pilot policy can increase the efficiency of emissions by improving the innovative effect of green technologies. Column (4) shows that carbon efficiency improvement can be achieved through the optimization of the regional industrial structure. From column (6), it can be noticed that increasing government financial support of science and technology can also prompt the efficiency of carbon emissions. The results demonstrate that the emission efficiency of cities is mainly improved through green technology effect, industrial structure effect, and government science and technology expenditure effect.

## 5. Spatial Effect Analysis

### 5.1. Spatial Econometric Model

As suggested by the existing literature, there is an extent of spatial correlation in pollution emissions between municipalities in China, which disrupts the results if ignored. Therefore, we adopt a spatial panel model to explore the policy effect on carbon efficiency in a certain region and adjacent areas under the condition of spatial spillover effect. The spatial econometric model is as follows:

(4) $$SCO_{2_{it}}=\alpha_{0}+\rho_{0}WSCO_{2_{it}}+\alpha_{1}du+\alpha_{2}dt+\alpha_{3}DID+\alpha_{4}WDID+\beta_{x}controls+\theta_{x}controls+\varepsilon_{it},$$

where W is the spatial weights matrix, measured by the inverse distance matrix, $\theta_{x}$ is the spatial regression coefficient of the control variable, $\rho_{0}$ is the spatial lag coefficient of the independent variable to be estimated, and other parameters are consistent with the model (1) definition.

### 5.2. Regression Results

Owing to the existence of regional spatial correlation, the spatial panel lag model (SAR) and Durbin model (SDM) were used to discuss the influence of pilot policy on regional carbon efficiency. First, the spatial relevance of carbon efficiency between regions is examined using the Moran index method. Moran index (Moran I) is calculated as follows:

(5) $$I=\frac{\sum_{i=1}^{n}\sum_{j=1}^{n}w_{ij}\left(x_{i}-\bar{x}\right)\left(x_{j}-\bar{x}\right)}{S^{2}\sum_{i=1}^{n}\sum_{j=1}^{n}w_{ij}},$$

where *n* is the number of samples, *n* = 281; $x_{i}$ and $x_{j},$ respectively, represent the GDP of the *i*th spatial unit and the *j*th spatial unit; $S^{2}$ represents the variance of *x* for 281 cities; $\bar{x}$ is the mean of *x* for 281 prefecture-level cities, and $w_{ij}$ is the spatial weight of the spatial weight matrix. From Table 11 and Table 12, it is noted that in the sample duration of 2006–2019, the global Moran Index (Moran I) of regional carbon emission efficiency was at least 1% significantly positive, illustrating that there was a remarkable favorable spatial relationship between carbon efficiency during the sample continuation duration, which further illustrates that bias in the estimation of research results will be led by the ignoration of spatial heterogeneity in innovative pilot policy, and the selection of spatial econometric models is reasonable and accurate.

**Figure 9:**
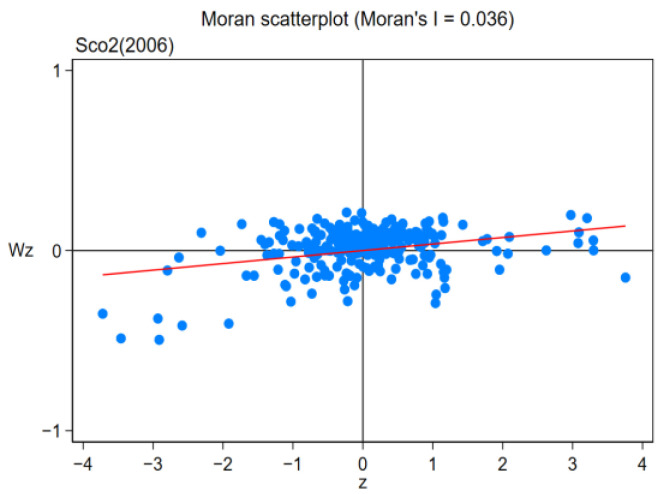
Moran Scatterplot (2006).

**Figure 10:**
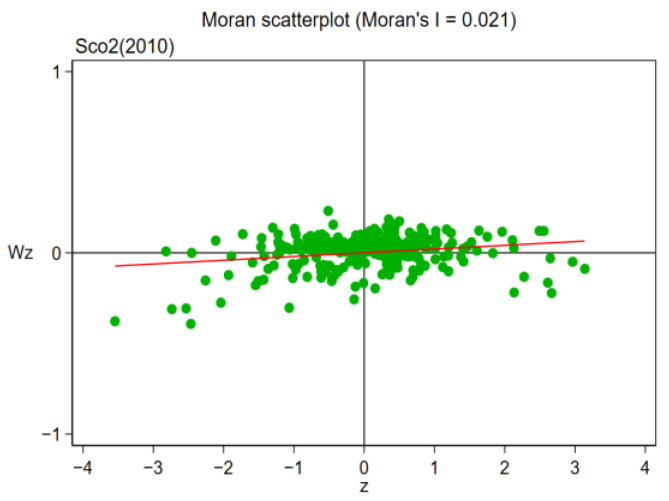
Moran Scatterplot (2010).

**Figure 11:**
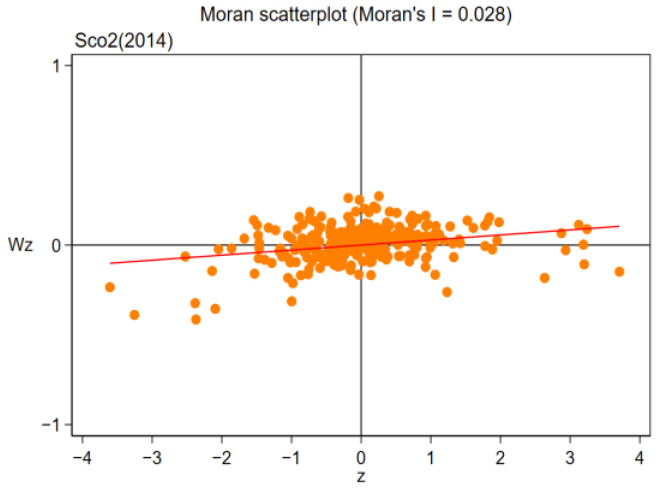
Moran Scatterplot (2014).

**Figure 12:**
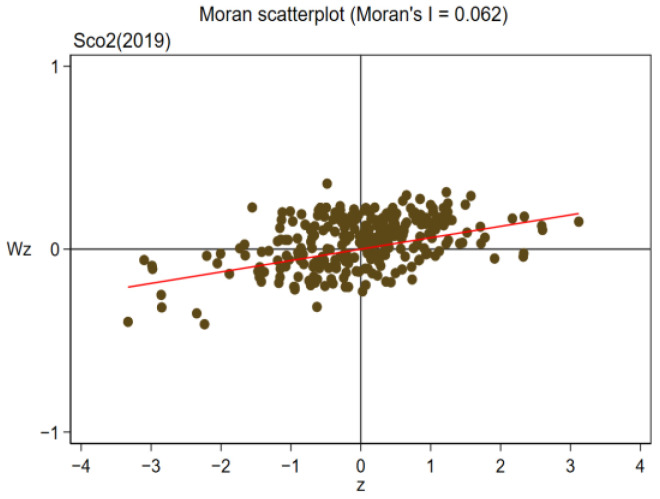
Moran Scatterplot (2019).

This paper further selects four years of 2006, 2010, 2014 and 2019 to draw Moran scatter plots. The specific results are shown in Figure 9, Figure 10, Figure 11 and Figure 12. As can be seen from the figure, the majority of cities are distributed in the first and third quadrants, showing agglomeration featured “low–low” or “high–high”, illustrating that the carbon efficiency of most cities is still at a poor stage.

Table 12 shows the results considering the spatial spillover effect, where columns (1) are the results of SAR under the inverse distance matrix, and columns (2) are the results of SDM under the inverse distance matrix. To examine the imitative effect of the spatial panel model selection, the Wald test and LR test were carried out on the basis of two spatial models, results of which were 34.51 and 34.12, respectively, and significant at least at the 1% level, showing that, compared with the SAR model, the SDM model had a better-fit effect in analyzing the reduction effect of the policy. The spatial lag term of the Durbin model is significantly positive, further demonstrating that there is an obvious relationship of carbon efficiency between regions.

The results of Table 12 are as follows. The coefficients of *Main DID* in the SAR model and the SDM model are both significantly positive, verifying that the innovative cities provide a favorable contribution to the promotion of carbon efficiency. The spatial lag term (*W-DID*) of the spatial Durbin model is 0.760, showing that the innovative cities have a positive spillover effect on the adjacent area, and the implementation of this policy in an area will significantly prompt the carbon emission performance of the adjacent area. For the direct effect, the innovative cities make a difference in promoting the carbon efficiency of adjacent regions, which confirms the robustness of the conclusion. For the indirect effect, both the coefficients under the SAR and SDM model are significantly positive. It can be concluded that the spillover effect generated by the innovative cities is significantly positive, and the innovative cities in the region make a big difference in promoting the carbon efficiency of adjacent regions. For the total effect, the local implementation of the innovative city pilot policy makes a significantly positive difference in increasing carbon emission efficiency of all regions, explaining that the urban innovative pilot policy can form a policy effect spillover, which is conducive to the formation of a demonstration effect of the policy and promotes the carbon efficiency of the neighboring areas.

## 6. Conclusions and Implications

### 6.1. Conclusions

We regard the innovative city pilot carried out in different cities and at different times as a quasi-natural experiment. By choosing a multi-period DID method, we explore the policy effect of innovative cities pilot on carbon efficiency. The findings suggest:

(1) The carbon emission efficiency of innovative pilot cities is noticeably improved, while the improvement of non-pilot cities is not obvious. The effect of innovative cities on carbon emission performance has been continuously enhanced over time. The conclusion demonstrated that innovative cities can play a favorable role in promoting the performance of urban carbon emission, which remains robust after a range of tests such as the exclusion of interference policy, addition of covariates, and winsorize.
(2) There is heterogeneity between different categories of cities, that is, the influence of the pilot city on non-resource cities and environmentally friendly cities are more significant from the perspective of different categories of cities.
(3) What is revealed by the Mechanism analysis is that the innovative cities improve carbon emission performance by reducing carbon emission levels, which is attributed to the fact that the innovative cities can strengthen urban green technology innovation, increase government financial support, and optimize the urban industrial structure.
(4) There is a space emission reduction effect of the pilot cities. The innovative city will markedly prompt the carbon performance of the adjacent regions.

The shortcomings of this paper are as follows: first, this paper only focuses on the carbon emission reduction effect of cities at the macro level. Future studies can explore the emission reduction effect of national innovative city pilot policies from the micro perspective of enterprises. Second, this paper only discusses the impact of a single policy of innovative city pilot policy on carbon emission efficiency, and the subsequent research can investigate the linkage effect between different policies.

### 6.2. Implications

In the light of above findings, this paper presents three recommendations:

(1) Fully prompt and expand the polit scope of innovative city. All levels of government should sum up the lessons learned during the process of the pilot policy and combine the current economic development and the current pollution emission situation of the region to promote the construction of China’s innovative cities as a whole while ensuring economic development. Through the comprehensive implementation of innovative urban construction, the pollution emission problems of various cities will be significantly improved, and the green development of the economy will be realized.
(2) Explore the road of independent innovation in the city and elevate the ability of independent Rand D ability. First of all, all levels of government can increase government capital expenditure to build innovation cooperation and exchange platform, promote the agglomeration of urban technology elements and innovation elements. Second, fully mobilize the coordination mechanism for the coordinated development of industries. All levels of governments should adjust the inherent model of industrial development to cultivate new competitive advantages and explore new momentum for regional economic development. Finally, adhere to the principle of government guidance in promoting innovation pilot work. Local governments should play a leading role, expand local investment expenditure, and strengthen the construction of environmental protection infrastructure. Increase expenditure on science and technology education and support for scientific research to units, and cultivate high-tech talents.
(3) It is essential to fully consider the spatial emission reduction effect of the pilot cities on adjacent areas. Therefore, the central government should optimize the spatial layout of innovative city construction and take the strategy of point to an area and maximize the influence of pilot cities on carbon performance. Simultaneously, differentiated strategies should be formulated to avoid following suit and promote the balanced development and coordinated advancement of China’s innovative city pilot work.

## Figures and Tables

**Table 1:** Variable Descriptive Statistics.

*Type*	*Index*	*Symbol*	*Obs*	*Min*	*Mean*	*Sd*	*Max*
*dependent*	*emission efficiency*	*Sco2*	3934	−2.641	−1.104	0.403	0.599
*core independent*	*the status of innovative city* *(dummy variable)*	*DID*	3934	−1.969	−0.680	0.303	0.255
*control*	*population density*	*popden*	3934	1.609	5.729	0.775	7.882
	*industrial structure*	*industry*	3934	11.70	47.84	10.94	90.97
	*FDI proportion*	*fdi*	3934	4.317	13.29	1.873	18.83
	*gross domestic product per capita*	*pgdp*	3934	4.595	10.41	0.726	13.06
	*local budget revenue*	*finan*	3934	9.722	13.57	1.212	18.09

**Table 2:** Baseline Regression.

	(1)	(2)	(3)	(4)
	*Sco2*	*Sco2*	*Sco2*	*Sco2*
*DID*	0.137 ***	0.247 ***	0.189 ***	0.189 ***
	(0.019)	(0.018)	(0.019)	(0.018)
*_cons*	−1.121 ***	−1.136 ***	−1.128 ***	−4.668 ***
	(0.007)	(0.004)	(0.004)	(0.300)
*control*	*N*	*N*	*N*	*Y*
*City-fixed effect*	*N*	*Y*	*Y*	*Y*
*Year-fixed effect*	*N*	*N*	*Y*	*Y*
*N*	3934	3934	3934	3934
*R-sq*	0.013	0.701	0.722	0.756

Note: *** denotes significant value at the level of 1%.

**Table 3:** Replacing dependent variable.

	(1)	(2)	(3)	(4)
	*Eco2*	*Eco2*	*Eco2*	*Eco2*
*DID*	0.068 ***	0.118 ***	0.136 ***	0.139 ***
	(0.014)	(0.013)	(0.014)	(0.013)
*_cons*	−0.689 ***	−0.695 ***	−0.698 ***	−3.567 ***
	(0.005)	(0.003)	(0.003)	(0.225)
*control*	*N*	*N*	*N*	*Y*
*City-fixed effect*	*N*	*Y*	*Y*	*Y*
*Year-fixed effect*	*N*	*N*	*Y*	*Y*
*N*	3934	3934	3934	3934
*R-sq*	0.006	0.702	0.715	0.757

Note: *** denotes significant value at the level of 1%.

**Table 4:** PSM-DID.

	(1)	(2)	(3)	(4)
	*Radius Matching*		*Kernel Matching*	
	*Sco2*	*Sco2*	*Sco2*	*Sco2*
*DID*	0.185 ***	0.145 ***	0.184 ***	0.149 ***
	(0.022)	(0.020)	(0.021)	(0.020)
*_cons*	−1.111 ***	−6.453 ***	−1.110 ***	−6.500 ***
	(0.005)	(0.382)	(0.005)	(0.378)
*control*	*N*	*Y*	*N*	*Y*
*City-fixed effect*	*Y*	*Y*	*Y*	*Y*
*Year-fixed effect*	*Y*	*Y*	*Y*	*Y*
*N*	2993	2993	3043	3043
*R-sq*	0.728	0.771	0.726	0.769

Note: *** denotes significant value at the level of 1%.

**Table 5:** Excluding other policies.

	(1)	(2)	(3)	(4)	(5)	(6)
	*The Policy of* *Low-Carbon City Pilot*		*The Policy of* *Emissions Trading Right*		*The Pilot Policy of * *New Energy Vehicles*	
	*Sco2*	*Sco2*	*Sco2*	*Sco2*	*Sco2*	*Sco2*
*DID*	0.144 ***	0.133 ***	0.178 ***	0.166 ***	0.162 ***	0.158 ***
	(0.024)	(0.023)	(0.020)	(0.019)	(0.023)	(0.022)
*_cons*	−1.137 ***	−5.528 ***	−1.158 ***	−4.687 ***	−1.134 ***	−4.712 ***
	(0.005)	(0.424)	(0.005)	(0.319)	(0.004)	(0.309)
*control*	*N*	*Y*	*N*	*Y*	*N*	*Y*
*City-fixed effect*	*Y*	*Y*	*Y*	*Y*	*Y*	*Y*
*Year-fixed effect*	*Y*	*Y*	*Y*	*Y*	*Y*	*Y*
*N*	2632	2632	3304	3304	3612	3612
*R-sq*	0.726	0.763	0.702	0.745	0.711	0.746

Note: *** denotes significant value at the level of 1%.

**Table 6:** Adding covariates.

	(1)	(2)	(3)	(4)
	*Sco2*	*Sco2*	*Sco2*	*Sco2*
*DID*	0.073 ***	0.087 ***	0.133 ***	0.072 ***
	(0.021)	(0.021)	(0.022)	(0.021)
*_cons*	−3.091 ***	−6.013 ***	−1.338 ***	−6.093 ***
	(0.215)	(0.322)	(0.141)	(0.315)
*control*	*N*	*Y*	*N*	*Y*
*City-fixed effect*	*Y*	*Y*	*Y*	*Y*
*Year-fixed effect*	*Y*	*Y*	*Y*	*Y*
*N*	3934	3934	3934	3934
*R-sq*	0.756	0.776	0.751	0.782

Note: *** denotes significant value at the level of 1%.

**Table 7:** Robustness Test.

	(1)	(2)	(3)	(4)	(5)	(6)
	*Sco2*	*Sco2*	*Sco2*	*Sco2*	*Sco2*	*Sco2*
*DID*	0.189 ***	0.183 ***	0.126 ***	0.145 ***	0.188 ***	0.187 ***
	(0.018)	(0.018)	(0.019)	(0.019)	(0.019)	(0.018)
*_cons*	−1.128 ***	−5.543 ***	−1.127 ***	−3.739 ***	−1.132 ***	−4.641 ***
	(0.004)	(0.309)	(0.004)	(0.756)	(0.004)	(0.301)
*control*	*N*	*Y*	*N*	*Y*	*N*	*Y*
*City-fixed effect*	*Y*	*Y*	*Y*	*Y*	*Y*	*Y*
*Year-fixed effect*	*Y*	*Y*	*Y*	*Y*	*Y*	*Y*
*N*	3934	3934	2248	2248	3878	3878
*R-sq*	0.719	0.758	0.835	0.840	0.717	0.751

Note: *** denotes significant value at the level of 1%.

**Table 8:** Heterogeneity of Urban Resource Endowments.

	(1)	(2)	(3)	(4)
	*Non-Resource-Based City*		*Resource-Based City*	
	*Sco2*	*Sco2*	*Sco2*	*Sco2*
*DID*	0.209 ***	0.201 ***	0.021	0.028
	(0.021)	(0.021)	(0.045)	(0.042)
*_cons*	−1.097 ***	−5.854 ***	−1.182 ***	−3.518 ***
	(0.006)	(0.427)	(0.006)	(0.421)
*control*	*N*	*Y*	*N*	*Y*
*City-fixed effect*	*Y*	*Y*	*Y*	*Y*
*Year-fixed effect*	*Y*	*Y*	*Y*	*Y*
*N*	2520	2520	1414	1414
*R-sq*	0.719	0.753	0.722	0.765

Note: *** denotes significant value at the level of 1%.

**Table 9:** Heterogeneity of City Types.

	(1)	(2)	(3)	(4)
	*Non-Environmentally Friendly Cities*		*Environmentally Friendly Cities*	
	*Sco2*	*Sco2*	*Sco2*	*Sco2*
*DID*	0.116 **	0.113 **	0.193 ***	0.161 ***
	(0.042)	(0.041)	(0.021)	(0.018)
*_cons*	−1.103 ***	−4.245 ***	−1.172 ***	−7.003 ***
	(0.005)	(0.393)	(0.008)	(0.444)
*Control*	*N*	*Y*	*N*	*Y*
*City-fixed effect*	*Y*	*Y*	*Y*	*Y*
*Year-fixed effect*	*Y*	*Y*	*Y*	*Y*
*N*	2548	2548	1386	1386
*R-sq*	0.695	0.724	0.791	0.843

Note: ** and *** denote significant values at the level of 5% and 1%, respectively.

**Table 10:** Transmission Mechanism Test.

	(1)	(2)	(3)	(4)	(5)	(6)
	*inno*	*inno*	*stru*	*stru*	*tech*	*tech*
*DID*	0.073 **	0.093 **	0.121 ***	0.029 **	0.194 ***	0.180 ***
	(0.037)	(0.037)	(0.017)	(0.011)	(0.039)	(0.036)
*_cons*	4.405 ***	−1.469 **	0.899 ***	0.950 ***	9.793 ***	−2.492 ***
	(0.008)	(0.616)	(0.004)	(0.191)	(0.009)	(0.597)
*control*	*N*	*Y*	*N*	*Y*	*N*	*Y*
*City-fixed effect*	*Y*	*Y*	*Y*	*Y*	*Y*	*Y*
*Year-fixed effect*	*Y*	*Y*	*Y*	*Y*	*Y*	*Y*
*N*	3715	3715	3933	3933	3933	3933
*R-sq*	0.942	0.945	0.851	0.937	0.923	0.940

Note: ** and *** denote significant values at the level of 5% and 1%, respectively.

**Table 11:** 2006–2019 China Carbon Emission Efficiency Global Moran Index.

***Year***	**2006**	**2007**	**2008**	**2009**	**2010**	**2011**	**2012**
*Moran I*	0.036 ***	0.034 ***	0.036 ***	0.022 ***	0.021 ***	0.020 ***	0.018 ***
*p value*	0.000	0.000	0.000	0.000	0.000	0.000	0.000
***Year***	***2013***	***2014***	***2015***	***2016***	***2017***	***2018***	***2019***
*Moran I*	0.019 ***	0.028 ***	0.036 ***	0.051 ***	0.051 ***	0.049 ***	0.062 ***
*p value*	0.000	0.000	0.000	0.000	0.000	0.000	0.000

Note: *** denotes significant value at the level of 1%.

**Table 12:** Spatial Effect Test of the Innovative City Pilot Policy.

	(1)	(2)
	*SAR*	*SDM*
	*Sco2*	*Sco2*
*Main DID*	0.184 ***	0.170 ***
	(0.017)	(0.018)
*W-DID*		0.760 ***
		(0.193)
*Spatial rho*	0.506 ***	0.519 ***
	(0.091)	(0.095)
*Direct DID*	0.185 ***	0.177 ***
	(0.018)	(0.018)
*Indirect DID*	0.203 **	1.791 ***
	(0.091)	(0.510)
*Total DID*	0.388 ***	1.967 ***
	(0.097)	(0.512)
*control*	*Y*	*Y*
*City-fixed effect*	*Y*	*Y*
*Year-fixed effect*	*Y*	*Y*
*N*	*3934*	*3934*
*R-sq*	*0.024*	*0.006*

Note: ** and *** denote significant values at the level of 5% and 1%, respectively.

## Data Availability

The datasets used and analyzed in the current study are available from the corresponding author upon reasonable request.
